# Calophyllolide Content in *Calophyllum inophyllum* at Different Stages of Maturity and Its Osteogenic Activity

**DOI:** 10.3390/molecules200712314

**Published:** 2015-07-07

**Authors:** Wei-Hsien Liu, Yen-Wenn Liu, Zih-Fong Chen, Wen-Fei Chiou, Ying-Chieh Tsai, Chien-Chih Chen

**Affiliations:** 1 Institute of Biochemistry and Molecular Biology, National Yang-Ming University, No. 155, Li-Nong St., Sec. 2, Beitou Dist., Taipei 11221, Taiwan; E-Mail: liuliu83@yahoo.com.tw; 2 National Research Institute of Chinese Medicine, No. 155-1, Li-Nong St., Sec. 2, Beitou Dist., Taipei 11221, Taiwan; E-Mails: skywenn@gmail.com (Y.-W.L.); wfchiou@nricm.edu.tw (W.-F.C.); 3 Department of Biotechnology, HungKuang University, No. 1018, Sec. 6, Taiwan Boulevard, Shalu District, Taichung City 43302, Taiwan; E-Mail: chiling0620@yahoo.com.tw; 4 Department of Nursing, HungKuang University, No. 1018, Sec. 6, Taiwan Boulevard, Shalu District, Taichung City 43302, Taiwan

**Keywords:** *Calophyllum inophyllum*, calophyllolide, osteoblast

## Abstract

*Calophyllum inophyllum* is a coastal plant rich in natural substances. Its ingredients have been used for the development of an anti-human immunodeficiency virus (HIV) drug. In this study, we collected *C. inophyllum* fruit, and the ethanol extract of the fruit was chromatographically separated using silica gel and Sephadex LH-20 columns to obtain the major compound, calophyllolide. The fruits were harvested from September to December in 2011; a quantitative analysis of the calophyllolide content was conducted using HPLC to explore the differences between the different parts of the fruit during the growing season. The results showed that in fruits of *C. inophyllum*, calophyllolide exists only in the nuts, and dried nuts contain approximately 2 mg·g^−1^ of calophyllolide. The calophyllolide levels in the nuts decreased during maturity. In addition, calophyllolide dose-dependently enhanced alkaline phosphatase (ALP) activity in murine osteoblastic MC3T3-E1 cells, without significant cytotoxicity. The expression of osteoblastic genes, ALP and osteocalcin (OCN), were increased by calophyllolide. Calophyllolide induced osteoblasts differentiation also evidenced by increasing mineralization and ALP staining.

## 1. Introduction

*Calophyllum inophyllum* (*C. inophyllum*; Guttiferae) is a large evergreen tree that grows along the western coast of Taiwan. Since it is a tropical plant, it grows well in whole Taiwan after implanting. The blossom season of *C.* *inophyllum* is June to July and the fruiting season is August to December. The mature fruit of *C. inophyllum* is edible raw and as raw fruit pickled with sugar. Additionally, the seeds are used as a source of oil for dyes and lubricants. In Taiwan, the entire *C. inophyllum* plant is used as a folk medicine to treat eye diseases, rheumatoid arthritis, contusions, sprains, and fractures. The resin of *C. inophyllum* is used to treat odontalgia and gum bleeding. Phytochemical studies examining different parts of *C. inophyllum*, such as the leaves [1], branches [2], stem bark [3], roots [4], and seeds [5], have isolated and identified several compounds, including xanthones [6], steroids, triterpenoids [7], and coumarins [8,9]. Some of these isolated compounds have been reported to be biologically active, with cytotoxic [10], repellent [11], anti-inflammatory [12], anti-microbial [10], and anti-human immunodeficiency virus (HIV) [8,13] properties.

**Figure 1:**
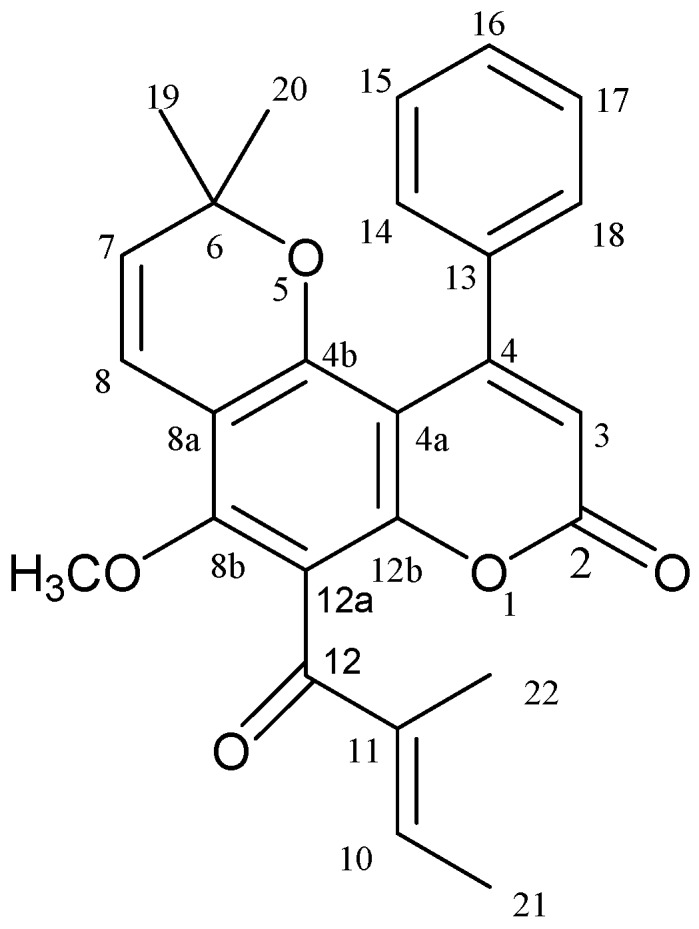
Structure of calophyllolide.

Calophyllolide (Figure 1) is the representative coumarin in *C. inophyllum*. Calophyllolide has been reported to exhibit some biological activity, including anti-inflammation, lower capillary vascular permeability [14], anti-cancer [15], anti-microbial [10], and anti-coagulant [9] properties.

Recently, we investigated a Taiwan herbal medicine which was used as fracture and contusion remedy for its effects on bone cells, osteoclasts and osteoblasts [16,17,18]. In this study, we isolated calophyllolide and studied the calophyllolide content in different parts of the *C. inophyllum* fruit at different maturation stages using HPLC. The effects of calophyllolide on osteoblasts, alkaline phosphatase (ALP) activity and mineralization, were also investigated. Gene expression of molecules involved in osteoblastdifferentiation, including ALP, runt related gene 2 (RUNX2), osterix (OSX), and osteocalcin (OCN) were also analyzed.

## 2. Results and Discussion

### 2.1. Calophyllolide Isolated from C. inophyllum Nuts

*C. inophyllum* fruits were collected from September to December in Taichung City, Taiwan. Ethanol extracts of the fruits were chromatographically separated multiple times on silica gel and Sephadex LH-20 columns to obtain pure calophyllolide (Figure 1). The structure of calophyllolide was confirmed using spectroscopic methods (^1^H-NMR and MS), and by comparison with published data in the literature [19]. The purity of the calophyllolide was greater than 98%, according to the NMR spectra and HPLC profile.

### 2.2. Quantification of the Calophyllolide in Nuts

**Figure 2:**
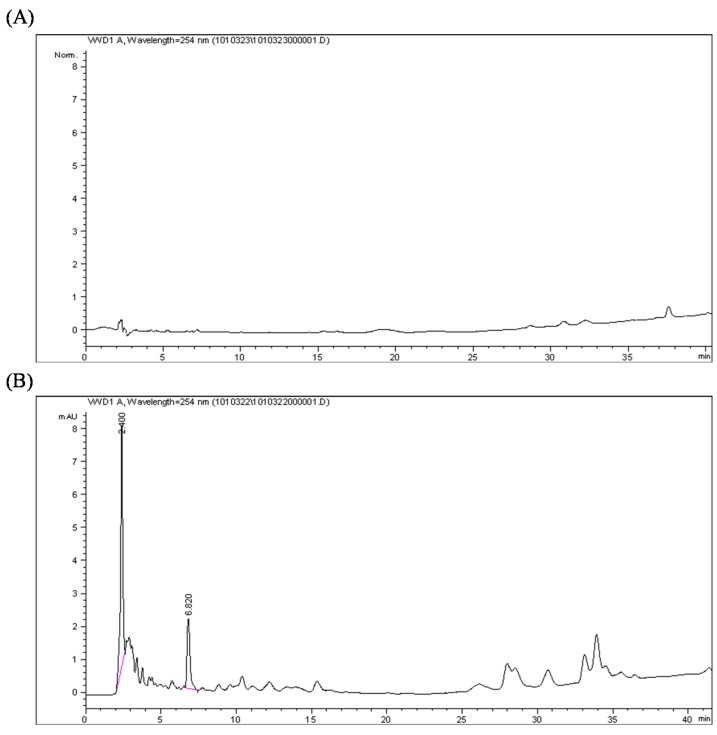

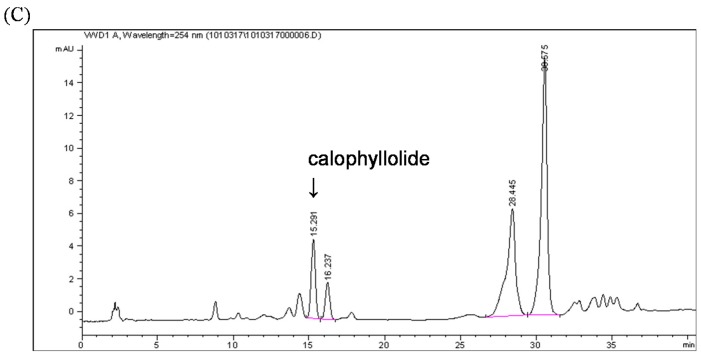
HPLC chromatograms of the (**A**) peel; (**B**) shell; and (**C**) nut of *Calophyllum inophyllum*, recorded at 254 nm. The retention time of calophyllolide is 15 min.

Calophyllolide is the major compound in different parts of *C. inophyllum*, including the seeds [5,20] and leaves [21]. By analyzing ethanolic extracts of different parts of *C. inophyllum* fruits, we found that the nuts are rich in calophyllolide, but almost no calophyllolide was found in the peel and nut shells (Figure 2).

**Figure 3:**
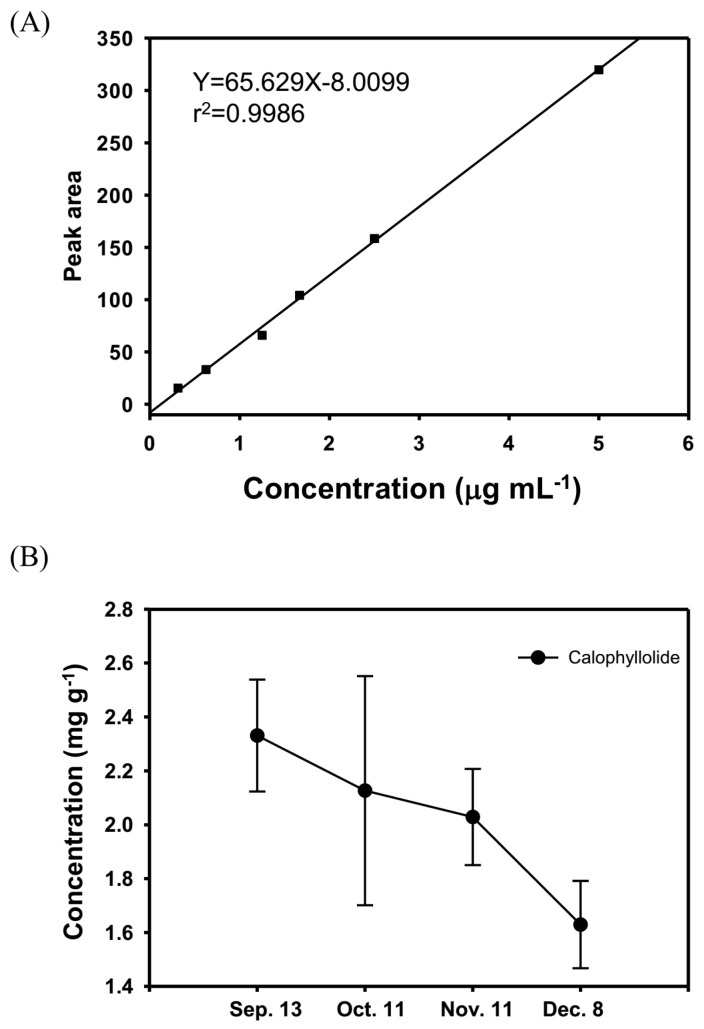
The calophyllolide content in nuts at different stages of maturity. (**A**) The HPLC standard curve of calophyllolide for quantification; (**B**) The contents of calophyllolide in nuts of *C. inophyllum* during maturation (from 13 September to 8 December). The data are shown as the mean ± SD.

The calophyllolide content in nuts at different stages of maturity was analyzed by HPLC. The calophyllolide content in dried nuts was approximately 2.3 mg·g^−1^ in samples collected in September, whereas in samples collected in December, the calophyllolide content decreased to 1.6 mg·g^−1^. Our results showed that the greater the maturity of the fruit, the less calophyllolide is found in nuts of *C. inophyllum* (Figure 3).

### 2.3. Calophyllolide-Induced Osteoblast MC3T3-E1 Cell Differentiation

**Figure 4:**
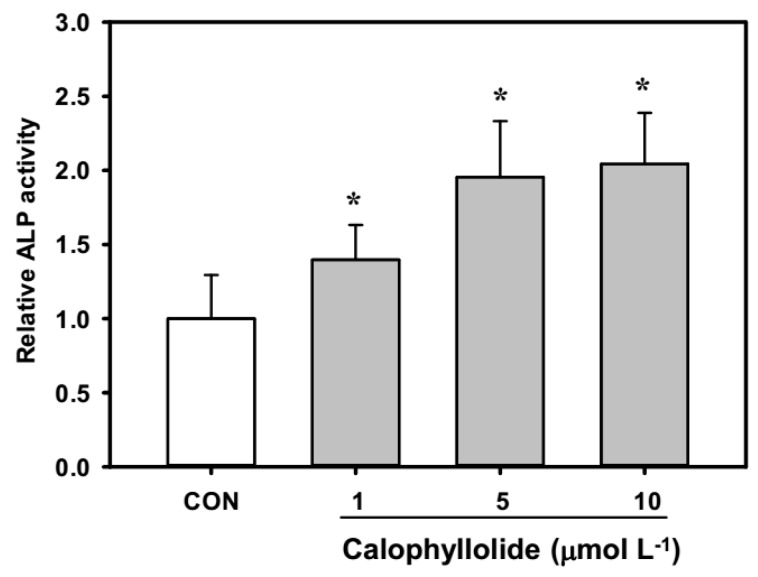
Alkaline phosphatase (ALP) activity of calophyllolide-treated MC3T3-E1 cells. The data are shown as the mean ± SD (with three replicates). The differences between comparison groups were considered statistically significant when *p* < 0.05 (*).

*C. inophyllm* is traditionally used in Taiwan as a folk medicine for the treatment of fractures and contusions. However, the effects of calophyllolide on bone-related cells have not previously been investigated. This is the first report to show that calophyllolide increased the alkaline phosphatase (ALP) activity in MC3T3-E1 cells in a dose-dependent trend (1, 5, and 10 μmol·L^−1^). Calophyllolide dose-dependently induced osteoblastic differentiation by significantly enhancing ALP activity (Figure 4; *p* < 0.05). The cell viability was comparable in all calophyllolide treated cells (data not shown).

### 2.4. Calophyllolide Induced Osteoblastic Gene Expression in MC3T3-E1 Cells

**Figure 5:**
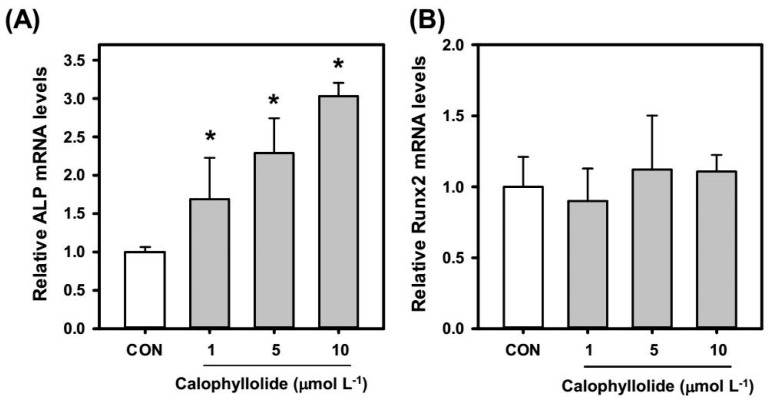

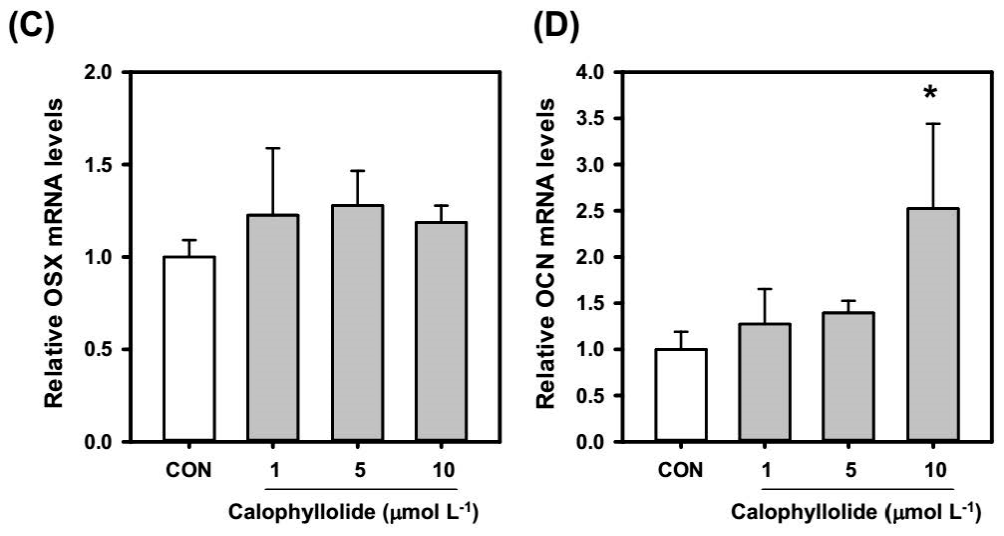
Effects of calophyllolide on osteogenic mRNA expression in MC3T3-E1 cells. After treated cells with calophyllolide (1, 5, and 10 μmol·L^−1^) for 4 days, total RNA was prepared for quantitative real-time PCR analysis. The mRNA levels (2^−ΔCt^) of (A) alkaline phosphatase (ALP); (**B**) runt related gene 2 (RUNX2); (**C**) osterix (OSX); and (**D**) osteocalcin (OCN) were determined using quantitative real-time PCR and calculated by subtracting the Ct value of GAPDH from the Ct value of the target gene (ΔCt = Ct_target_ − Ct_GAPDH_). The values are represented as folds of untreated control group (CON). The data are expressed as the mean ± SD (with three replicates). A difference between calophyllolide-treated group and CON was considered statistically significant when *p* < 0.05 (*).

Calophyllolide treatment significantly induced ALP activity in MC3T3-E1 cells which suggested the osteogenic effects of calophyllolide. We further investigated the expression of osteoblastic genes, ALP, RUNX2, OSX, and OCN. According to our results, the mRNA expression levels of ALP (Figure 5A) in calophyllolide-treated MC3T3-E1 cells were dose-dependently increased. Calophyllolide didn’t affect the expression of RUNX2 and OSX mRNA (Figure 5B,C).

Furthermore, the mRNA expression of OCN was increased when MC3T3-E1 cells were treated with calophyllolide at 10 μmol·L^−1^ (Figure 5D).

### 2.5. Calophyllolide Enhanced Mineralization and ALP Expression of MC3T3-E1 Cells

**Figure 6:**
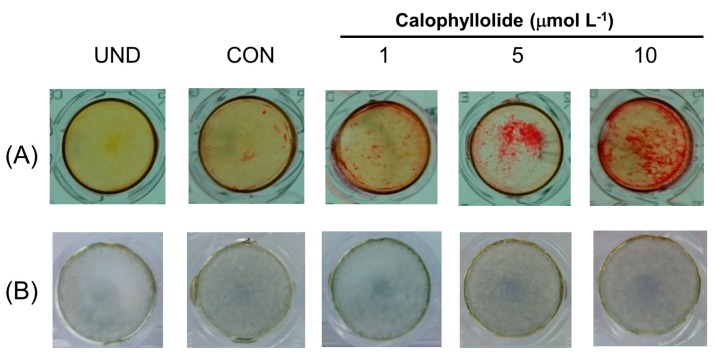
Effects of calophyllolide on mineralization and ALP stain of MC3T3-E1 cells. After treated MC3T3-E1 cells with calophyllolide (1, 5, and 10 μmol·L^−1^), Alazarin red-S staining procedure (**A**) and ALP staining procedure (**B**) were used to investigate the effects of calophyllolide on MC3T3-E1 cells.

Mineralization assay is used to investigate the maturation of osteoblasts by detecting the bone nodules formation of osteoblasts during osteoblastic differentiation. As shown in Figure 6A, calophyllolide treatment significantly enhanced the bone nodules formation in MC3T3-E1 cells compared with control cells on day 18.

The ALP expression in differentiated MC3T3-E1 cells-treated with calophyllolide was analyzed by ALP stain. Calophyllolide-treated cells showed more ALP-stained spot which suggested calophyllolide increased the ALP protein expression in MC3T3-E1 cells (Figure 6B).

### 2.6. Discussion

The present study examined the fruits of *C. inophyllum* collected at different stages of maturity, and ethanol extracts of the fruits were chromatographically separated using silica gel and Sephadex LH-20 to afford calophyllolide (Figure 1) [20]. The structure of calophyllolide was verified using spectroscopic methods, ^1^H-NMR and MS, and by comparison with data published in the literature.

Calophyllolide is a constituent of *C. inophyllum* and has been reported to have some biological activity. Bhalla *et al.*, reported calophyllolide to be a non-steroidal anti-inflammatory agent [22]. The ability of calophyllolide to decrease capillary permeability was reported later [14]. Calophyllolide, along with other compounds isolated from *C. inophyllum*, has been reported to exhibit anti-microbial and cytotoxic activities [10]. Calophyllolide has also been reported to induce apoptosis in HL-60 leukemia cells via caspase-9/caspase-3 activation [15]. However, the effect of calophyllolide on bone-related cells, osteoclasts and osteoblasts, has not previously been reported. Our team investigated Taiwan originated herbal medicine, which are used to treat fracture and contusion, for their effects on bone cells, osteoclasts and osteoblasts [16,17,18]. In view of the usage of *C. inophyllum* as fracture remedy, in the present study, we investigated the effects of calophyllolide on murine osteoblastic MC3T3-E1 cells. Our results showed that calophyllolide (30 μmol·L^−1^) significantly induced ALP activity, which is the early marker molecule of osteoblastic differentiation, in MC3T3-E1 cells. This is the first report to demonstrate that calophyllolide can induce osteoblastic cell (MC3T3-E1) differentiation. The current results suggest that the fruits of *C. inophyllum* could be a potential source for developing an anti-osteoporosis remedy.

Phytochemical profiles are considered to change during the maturation and ripening processes. The content of lycopene and citrulline in watermelon has been reported to increase upon ripening [23], which suggests an increase in nutritional value. In one study, the vitamin C content in guava fruit increased as the fruit matured, which can be explained by the breakdown of starch to glucose; this process increases the biosynthesis of ascorbic acid. However, the phenolic content in guava fruit decreased with maturation. A possible reason is that different phenolic acids might have condensed to form complex phenolic compounds, such as tannins and lignin, in the late stages of ripening [24]. As for palm fruit, the ferulic acid and caffeic acid content was reported to decrease during maturation because these substances are progressively bound to the cell walls [25]. In the present study, the calophyllolide content, which was only observed in *C. inophyllum* nuts, decreased with fruit maturation. Yimdjo *et al.* reported the antimicrobial and cytotoxic components from *C. inophyllum*. They found the content of calophyllolide in dried nuts is 0.94 mg·g^−1^ [10]. Laure *et al.*, screened inophyllums for anti-HIV activity from leaf extracts of *C. inophyllum* and the mean content of calophyllolide in leaf extracts is 1.93 ± 1.7 mg·kg^−1^ [21]. According to our results, the contents of calophyllolide in nuts are 1.6–2.3 mg·g^−1^. The result is similar to the report of Yimdjo *et al.* Furthermore, calophyllolide content in nut of *C. inophyllum* is significantly higher than in other parts of the plant. However, according to our result, the content of calophyllolide decreased during the fruit maturation, which suggests that calophyllolide might be transformed to other compounds. Further studies are needed to elucidate the mechanisms involved.

Calophyllolide is a coumarin compound. There are several reports regarding the effects of coumarins on MC3T3-E1. Coumarins isolated from *Artemisia iwayomogi* were reported to enhance osteoblast differentiation [26]. Two coumarin derivatives, imperatorin and bergapten, were reported to increase bone morphogenic protein (BMP)-2 expression and to enhance bone formation in murine primary osteoblasts [27]. Psoralen was reported to induce osteoblast differentiation via BMP signaling pathways [28]. Fraxetin, which is also a coumarin, induced the maturation and differentiation of the human osteoblast-like cell lines MG-63 and hFOB via the BMP-2 and BMP-4 pathways [29]. Osthole, a coumarin-like compound, was reported to induce osteoblast differentiation through β-catenin-BMP signaling [30]. In our results, calophyllolide-treated MC3T3-E1 cells showed increased ALP activity, which is an early marker of osteoblasts, suggesting that calophyllolide can induce osteoblast differentiation. We further investigated the mRNA expression of ALP. As shown in Figure 5A, the mRNA expression of ALP in calophyllolide-treated MC3T3-E1 cells was significantly and dose-dependently increased (*p* < 0.05) which is consistent with the ALP activity assay (Figure 4). OCN is produced by osteoblasts to be the most abundant proteins in bone. OCN is believed to be a serum marker of osteoblastic bone formation and acts in the bone matrix then regulates mineralization [31]. Calophyllolide stimulated the mRNA expression of OCN (Figure 5D) and also enhanced the mineralization of MC3T3-E1 cells (Figure 6A). The expression of RUNX2 and OSX is regulated by BMP-2. As for OSX, it is expressed in developing bones and is essential for osteoblast differentiation and bone formation [32]. In our results, the mRNA expression of RUNX2 and OSX are not affected by calophyllolide (Figure 5B,C). This may be due to calophyllolide didn’t alter the expression of BMP-2 mRNA (data not shown). The detail mechanisms for calophyllolide to induce osteoblasts differentiation needed further studies. Taken together, our results suggested that calophyllolide can induce osteoblasts differentiation by stimulating the expression of osteogenic genes, ALP and OCN. This is the first report to investigate the effects of calophyllolide on osteoblasts. Furthermore, the increase in ALP activity and ALP mRNA expression caused by calophyllolide suggests osteoblasts differentiation was induced by calophyllolide.

## 3. Experimental Section

### 3.1. General Information

^1^H, ^13^C, and 2D NMR spectra were recorded on a Varian VNMRS 600 MHz spectrometer (Varian, Palo Alto, CA, USA). ESIMS spectrum was obtained on Finnigan LCQ spectrometers (Thermo Finnigan LCQ-Duo, San Jose, CA, USA). HPLC was conducted on a HP model 1100 system (Angilent, Waldbronn, Germany) equipped with a HP G1311A QuatPump, a HP G1322A degasser, and a HP G1315B photodiode array detector set at 254 nm.

### 3.2. Plant Material

Fruit from *C. inophyllum* was collected four times from September to December in 2011 near the Long Jing interchange of Taichung City.

### 3.3. Chemicals

Sephadex LH-20 was purchased from Amersham Bioscience (Uppsala, Switzerland). Alizarin red-S, 2-amino-2-methyl-1-propanol (AMP), ascorbic acid, *N*,*N*-dimethylformamide, fast blue BB salt, naphthol AS-MX phosphate, *p*-nitrophosphate, and magnesium chloride (MgCl_2_) were purchased from Sigma Aldrich (St. Louis, MO, USA). β-Glycerophosphate was obtained from Wako Pure Chemicals (Osaka, Japan). α-Minimum essential medium (α-MEM), fetal bovine serum (FBS) and antibiotics (penicillin and streptomycin) were purchased from Gibco (Gibco BRL, Grand Island, NY, USA). Silica gel (60 N, 100–200 mesh) and other chemicals were purchased from Merck (Darmstadt, Germany). 

### 3.4. Calophyllolide Isolation, Purification and Identification

Freshly collected *C. inophyllum* fruits were cut up and homogenized using a lab blender, then extracted twice with ethanol to obtain crude ethanol extracts. The crude ethanol extracts were partitioned with water:ethyl acetate (1:1) to afford ethyl acetate extracts and water extracts. Calophyllolide was obtained from ethyl acetate extracts by separation on a silica gel column (solvent condition, *n*-hexane:EtOAc = 6:1) and Sephadex LH-20 (solvent condition, 100% MeOH).

### 3.5. Calophyllolide

^1^H-NMR (600 MHz, CDCl_3_): δ 0.94 (6H, s, H_3_-19 and H_3_-20), 1.86 (3H, d, *J* = 6.6 Hz, H_3_-21), 1.97 (3H, s, H_3_-22), 3.72 (3H, s, 8b-OCH_3_), 5.45 (1H, d, *J* = 9.6 Hz, H-7), 5.99 (1H, s, H-3), 6.42 (1H, d, *J* = 9.6 Hz, H-8), 6.54 (1H, q, *J* = 6.6 Hz, H-10), 7.21 (2H, m, H-14 and H-18), 7.36 (3H, m, H-15, H-16, and H-17); ^13^C-NMR (150 MHz, CDCl_3_): δ 10.7 (C-22), 15.2 (C-21), 26.9 (C-19 and C-20), 63.0 (C-OCH_3_), 105.6(C-4a), 110.7 (C-8a), 114.2 (C-3), 115.0 (C-12a), 115.9 (C-8), 127.2 (C-14 and C-18), 127.4 (C-15 and C-17), 127.7 (C-16), 129.0 (C-7), 139.5 (C-13), 139.9 (C-11), 144.2 (C-10), 151.7 (C-4b), 152.0 (C-12b), 155.0 (C-4), 155.8 (C-8b), 159.5 (C-2), 194.3 (C-12); ESIMS *m*/*z* 439 [M + Na]^+^, 417 [M + H]^+^.

### 3.6. Quantitative Analysis of Calophyllolide in the Fruits of C. inophyllum

Quantitative HPLC analysis was carried out to analyze the calophyllolide content in nuts at different maturation stages.

#### 3.6.1. Preparation of the Calophyllolide Standard Solution

To prepare calophyllolide standard solution, a calophyllolide standard (5 mg) was weighed precisely, and methanol (10 mL) was added. The solution was then filtered through a 0.22-μm membrane to yield a standard solution for HPLC analysis.

#### 3.6.2. Preparation of the *C. inophyllum* Nut Sample Solutions

The fruits of *C. inophyllum* were collected at different maturation stages. The collected *C. inophyllum* nuts were freeze-dried and then crushed. The crushed nuts (1 g) were extracted with ethanol (25 mL) twice. The ethanol extract was filtered with filter paper and then evaporated. After evaporation, 10 mL of MeOH was added to the extract, and the resulting solution was filtered with a 0.22 μm membrane for HPLC analysis. Each nut sample solution was prepared in triplicate.

#### 3.6.3. HPLC Analysis

A Cosmosil 5C18-AR-II (5 μm, 4.6 × 250 mm) column was used to analyze the calophyllolide content in the nut samples. A μBondpak C18 (Millipore, Milford, MA, USA) was used as a pre-column. To elute calophyllolide at ambient temperature, a gradient of water (solvent A) and acetonitrile (solvent B) was used. The eluent gradient program was as follows (A to B ratio is indicated): 0–10 min (30:70, *v*/*v*); 10–20 min (30:70, *v*/*v*); and 20–40 min (0:100, *v*/*v*). The flow rate was 1 mL/min. A UV detector at 254 nm was used to detect the signal.

### 3.7. Cell Culture and Differentiation of MC3T3-E1 Cells

The mouse osteoblast cell line MC3T3-E1 was purchased from the American Type Culture Collection (ATCC, Manassas, VA, USA) and maintained in α-minimum essential medium (α-MEM) supplemented with 10% fetal bovine serum (FBS) and antibiotics (penicillin 100 U·mL^−1^ and streptomycin 100 μg·mL^−1^) in a humidified atmosphere of 5% CO_2_ at 37 °C. For differentiation, 96-well plates were seeded with 10^4^ cells/well and incubated for 48 h. Then, cells were treated with calophyllolide (1, 5 and 10 μmol·L^−1^) in differentiation medium (α-MEM containing 10% FBS, 50 μg·mL^−1^ ascorbic acid and 10 mmol·L^−1^ β-glycerophosphate) for 3 days.

### 3.8. Alkaline Phosphatase (ALP) Assay

The ALP activity assay was performed according to a published method [33] with slight modifications. Briefly, after 3 days of incubation, the cells were washed twice with cold PBS and lysed by adding 0.01% SDS. The protein concentrations in the cell lysates were determined using Bio-Rad Dc protein assay reagents. The cell lysates were then added to 0.1 mol·L^−1^ NaHCO_3_-Na_2_CO_3_ buffer (pH 10) containing 5% AMP, 2 mmol·L^−1^ MgCl_2_, and 6 mmol·L^−1^*p*-nitrophenyl phosphate and allowed to incubate at 37 °C for 1 h. The reaction was stopped by adding 1 mol·L^−1^ NaOH, and the absorbance was measured at 405 nm. The ALP activity was normalized to the protein concentration in each cell lysate, and the value of the vehicle control group was defined as 1.

### 3.9. Quantitative Real-Time RT-PCR

After treated MC3T3-E1 cells with calophyllolide for 3 days, total RNA of treated-cells were prepared using the TRIzol method (Invitrogen, Carlsbad, CA, USA), and cDNA was then synthesized using the First Strand cDNA Synthesis Kit (Thermo Scientific, Waltham, MA, USA). Quantitative real-time PCR (qRT-PCR) was performed in a Roche LC-480 Real time PCR instrument according to the manufacturer’s recommendations. Primer sets and annealing temperatures are listed in Table 1. The housekeeping gene glyceraldehyde-3 phosphate dehydrogenase (GAPDH) was used as an internal control. The expression levels of target mRNAs of each sample were normalized to GAPDH as an internal control.

**Table 1:** Primer sets and annealing temperature for qRT-PCR.

Gene Name	Primer Sequence	Size (bp)	Annealing Temperature (°C)	Accession No.
ALP	F: 5′-TGACCTTCTCTCCTCCATCC-3′	126	55	NM_001287176.1
	R: 5′-CTTCCTGGGAGTCTCATCCT-3′			
RUNX2	F: 5′-CCCTGAACTCTGCACCAAGT	147	55	NM_001145920.2
	R: 5′-TGGAGTGGATGGATGGGGAT			
OSX	F: 5′-GTCAAGAGTCTTAGCCAAACTC-3′	124	55	NM_130458.3
	R: 5′-AAATGATGTGAGGCCAGATGG-3′			
OCN	F: 5′-CAATAAGGTAGTGAACAGAC-3′	133	50	NM_001305448.1
	R: 5′-CTTTAAGCCATACTGGTTT-3′			
GAPDH	F: 5′-GTATGACTCCACTCACGGCAAA-3′	101	60	NM_008084
	R: 5′-GGTCTCGCTCCTGGAAGATG-3′			

ALP, alkaline phosphatase; RUNX2, runt related gene 2; OSX, osterix; OCN, osteocalcin; GAPDH, glyceraldehyde-3 phosphate dehydrogenase. F, forward; R, reverse.

### 3.10. Mineralized Matrix Formation Assay and ALP Staining

Mineralization of MC3T3-E1 cells was measured by Alizarin red-S staining of calcium according to a published report [33]. MC3T3-E1 cells were seeded in 48-well culture plate at a density of 3 × 10^4^ cells/well and cultured for 2 days. On day 3, the cells were treated with calophyllolide (1, 5, and 10 μmol·L^−1^) in differentiation medium for a further 15 days with the medium and calophyllolide changed every 3 days. On day 18, the cells were washed with phosphate-buffered saline (PBS) and fixed with ice cold 75% EtOH for 30 min. Calcium deposits were stained using Alizarin-red S (40 mmol·L^−1^, pH 4.2) at room temperature for 15 min and examined under light microscope. For ALP staining observation, the cells were fixed with 3.7% formaldehyde at room temperature for 10 min after treating cells with calophyllolide for 18 days. After then, the cells were washed with PBS and incubated with ALP staining solution (0.1 mg·mL^−1^ of naphthol AS-MX phosphate, 0.5% *N*,*N*-dimethylformamide, 2 mmol·L^−1^ MgCl_2_, and 0.6 mg·mL^−1^ of fast blue BB salt in 0.1 mol·L^−1^ Tris-HCl, pH 8.5) at room temperature for 30 min [34].

### 3.11. Statistical Analysis

The results are expressed as the mean ± standard deviation (S.D.) of three replications. The differences between the mean values were tested for statistical significance using one-way ANOVA followed by Tukey’s *post-hoc* test. A *p*-value of <0.05 was considered statistically significant.

## 4. Conclusions

The current results revealed that calophyllolide from *C. inophyllum* nuts is an osteogenic compound and suggested that *C. inophyllum* nuts, which are rich in this compound, are a suitable material for developing osteogenic substances.
